# A novel artificial vertebral implant with Gyroid porous structures for reducing the subsidence and mechanical failure rate after vertebral body replacement

**DOI:** 10.1186/s13018-023-04310-6

**Published:** 2023-11-03

**Authors:** Peng Shang, Benyuan Ma, Guanghui Hou, Yihai Zhang, Lunxu Cui, Wanzhen Song, Yancheng Liu

**Affiliations:** 1 School of Mechanical Engineering, Hebei University of Technology, Tianjin, China; 2 Department of Bone and Soft Tissue Oncology, Tianjin Hospital, Tianjin, China

**Keywords:** Triply periodic minimal surface, Artificial vertebral implant, Prosthetic reconstructions, Finite element analysis, Selective laser melting

## Abstract

**Background:**

Prosthesis subsidence and mechanical failure were considered significant threats after vertebral body replacement during the long-term follow-up. Therefore, improving and optimizing the structure of vertebral substitutes for exceptional performance has become a pivotal challenge in spinal reconstruction.

**Methods:**

The study aimed to develop a novel artificial vertebral implant (AVI) with triply periodic minimal surface Gyroid porous structures to enhance the safety and stability of prostheses. The biomechanical performance of AVIs under different loading conditions was analyzed using the finite element method. These implants were fabricated using selective laser melting technology and evaluated through static compression and subsidence experiments.

**Results:**

The results demonstrated that the peak stress in the Gyroid porous AVI was consistently lower than that in the traditional porous AVI under all loading conditions, with a maximum reduction of 73.4%. Additionally, it effectively reduced peak stress at the bone-implant interface of the vertebrae. Static compression experiments demonstrated that the Gyroid porous AVI was about 1.63 times to traditional porous AVI in terms of the maximum compression load, indicating that Gyroid porous AVI could meet the safety requirement. Furthermore, static subsidence experiments revealed that the subsidence tendency of Gyroid porous AVI in polyurethane foam (simulated cancellous bone) was approximately 15.7% lower than that of traditional porous AVI.

**Conclusions:**

The Gyroid porous AVI exhibited higher compressive strength and lower subsidence tendency than the strut-based traditional porous AVI, indicating it may be a promising substitute for spinal reconstruction.

## Introduction

Spinal reconstruction poses a persistent challenge after partial corpectomy. While traditional techniques like titanium mesh cage (TMC) reconstruction are effective, they are associated with various issues, such as inadequate conformity to endplate shape and sagittal spine alignment, susceptibility to subsidence, and mechanical failure [1]. The application of 3D-printed artificial vertebral implants (AVIs) has received widespread attention in recent years. It can achieve precise alignment with adjacent endplates by computer scanning. The porous structures can reduce the elastic modulus of solid metal to be similar to the elastic modulus of human bone tissue, thus effectively reducing or eliminating the “stress shielding” effect [2–4]. Nevertheless, the majority of porous AVI currently employed in clinical practice utilizes strut-based traditional porous structures like body-centered cubic cells (BCC), face-centered cubic cells (FCC), and “diamond-like” structures [5–7], which suffer from inadequate compressive strength and propensity for stress concentration within their lattice structures [8–10]. Therefore, it is crucial to design novel implants with sound mechanical transmission and more uniform stress distribution than traditional porous vertebral implants.

Triply periodic minimal surface (TPMS) porous structures have zero-mean curvature at every surface point, enhancing their load-bearing capacity and mechanical properties. These structures exhibit a distinct morphology, allowing precise control and adjustment of morphological parameters like pore shape, size, strut thickness, and porosity. This enables adequate mechanical characteristics to sustain physiological loads and aligns with the specific mechanical demands of nearby bone tissue [11, 12]. There is growing interest in TPMS structures due to their increased biomechanical properties compared to their strut-based porous structures. While TPMS porous structures are currently employed in bone implants, such as femoral stems, with positive outcomes [13, 14], the biomechanical properties of TPMS porous AVIs have yet to be investigated.

The finite element method (FEM) was recognized as effective for evaluating the bone-implant system's biomechanical and mechanical properties. Simulating the normal physiological activities of the vertebra, such as flexion, extension, lateral bending, and axial rotation, comprehensively reflects a comprehensive performance of vertebral implants [15]. However, the porous structures are usually simplified as solid structures with equivalent mechanical properties to improve computational efficiency [16, 17]. This simplification overlooks the effect of the porous structures on stress transfer. It may lead to biased results that fail to accurately capture the stress distribution within the porous structures inside the implant. Therefore, establishing a high-precision finite element model of porous AVI becomes crucial for the biomechanical analysis of porous AVI.

Porous structures serve as an effective approach to reducing the stiffness of implants. However, conventional computer numerical control (CNC) machining faces challenges in fabricating such complex porous structures [18]. Selective laser melting (SLM) is a promising medical orthopedics manufacturing technique [19, 20]. This additive manufacturing (AM) process enables the construction of irregular and intricate three-dimensional porous metal parts by fusing fine metal powders [21]. While previous studies have conducted experimental studies on AM porous AVI to evaluate their mechanical properties [16, 22], more mechanical experiments on TPMS porous AVI still need to be conducted.

Consequently, a novel AVI with Gyroid porous structures was developed. Its biomechanical properties were evaluated using finite element and experimental methods to determine whether this novel AVI could reduce the subsidence and mechanical failure rate after vertebral body replacement. This study also provides a reference for the application and biomechanical analysis of TPMS porous structures in bone implants.

## Materials and methods

### Design of the TPMS Gyroid AVI

**Fig. 1:**
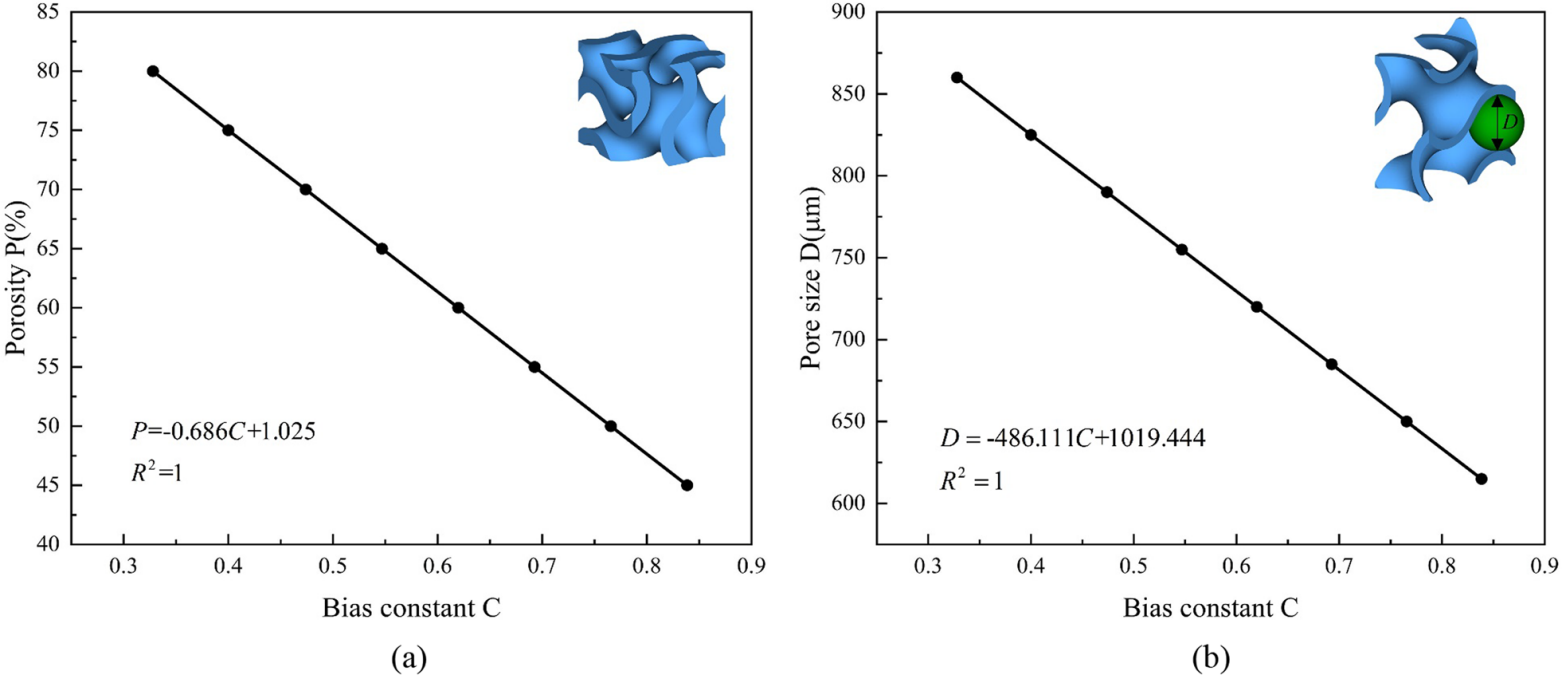
**a** The relationship between porosity *P* and bias constant *C*; **b** The relationship between pore size *D* and bias constant *C*

Various structures exist within the TPMS family, including Diamond, Neovius, wrapped package-graph (IWP), Schwarz Primitive, and Gyroid. While these structures belong to the same family, they often display distinct characteristics. The Gyroid structure is a representative example of TPMS porous structures. It faithfully reflects the architecture of various physical materials found in nature, including soap films and ultrastructures in butterflies [23, 24]. It has excellent mechanical strength and permeability compared to other TPMS porous structures. For instance, the uniaxial modulus, compressive strength, and energy absorption of the Gyroid structure have relatively good mechanical properties compared to the IWP, Neovius, and Primitive structures from a previous study [25]. Additionally, the Gyroid structures exhibited the highest permeability compared to the Diamond and Neovius structures with equivalent porosity [26]. Consequently, the Gyroid structure was selected among other TPMS and strut-based topologies due to its mechanical performance and proven versatility in multiple fields and applications [27–31]. The implicit surface equation of Gyroid structures is as follows [32]:

1 $$\cos \left( {\frac{2\pi }{b}x} \right)\sin \left( {\frac{2\pi }{a}y} \right) + \cos \left( {\frac{2\pi }{c}y} \right)\sin \left( {\frac{2\pi }{b}z} \right) + \cos \left( {\frac{2\pi }{a}z} \right)\sin \left( {\frac{2\pi }{c}x} \right) = C$$

where constants *a*, *b*, and *c* govern the unit cell size in three directions, the constant *C* controls the ratio of two volumes separated by the Gyroid surface and is termed bias constant in this study. The porosity *P* and pore size *D* of the Gyroid structure can be precisely adjusted by manipulating the bias constant *C*, allowing for quantitative tuning. This study discovered that when the dimensions *a*, *b*, and *c* are all 2 mm, the correlation between the bias constant *C* and the porosity *P* and the pore size *D* is illustrated in Fig. 1.

**Fig. 2:**
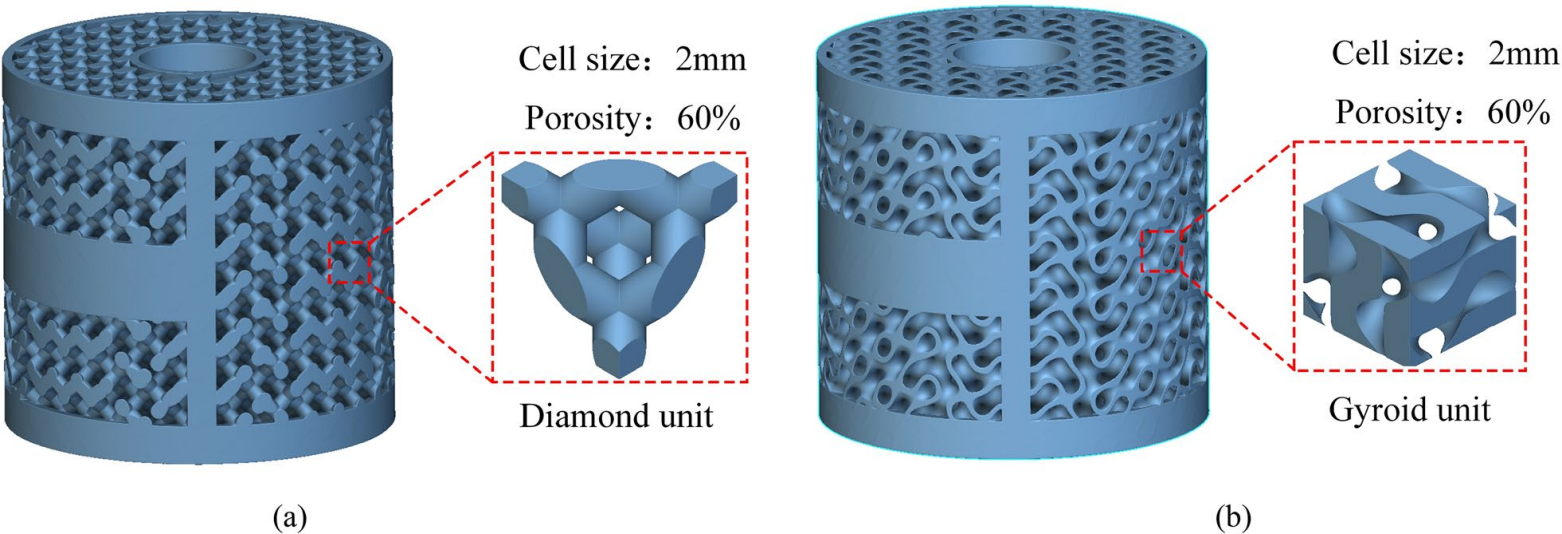
Porous AVI model and its cell parameters. **a** Traditional porous AVI; **b** Gyroid porous AVI

Porous structures that facilitate inward bone growth possess pore sizes ranging from 100 to 800 μm and a porosity exceeding 50% [33, 34]. Furthermore, research has demonstrated that a porosity of 60% is particularly favorable for promoting bone cell growth [19]. For this study, the Gyroid structure with a porosity of 60% was selected to design the porous region of the AVI. The pore size of the Gyroid structure is currently estimated to be around 725 μm, which meets the criterion for facilitating bone growth. The initial geometric model of the AVI is established based on the lesion's extent and the adjacent segments. Precisely, the height of the AVI corresponds to the extent of the patient's vertebral body resection. Additionally, the lordotic angle of the AVI was designed to be 0 degrees. To evaluate the biomechanical properties of TPMS Gyroid AVI, the traditional porous AVI consisting of trusses or beams was selected as the control group, and this type of implant has been applied in the clinic [7]. As shown in Fig. 2, the two kinds of AVIs have the same spatial contour, but their internal porous structures differ. The two porous structures are traditional “diamond-like” and TPMS Gyroid structures, representing strut- and sheet-based trabecular bone structures [35].

### Healthy finite element model

The data for constructing the L1–L5 lumbar spine FEM were obtained from a healthy adult male volunteer (26 years old, weight 85 kg, height 180 cm) with no history of trauma or fractures. The image data of five vertebrae from L1 to L5 were acquired using a 64-slice spiral computed tomography scanner (GE, Siemens Sensation 16 Slice, Germany) with an inter-layer spacing of 0.625 mm in DICOM format. The DICOM images were imported into Mimics 20.0 software (Materials Company, Leuven, Belgium) to generate a three-dimensional (3D) surface model of the vertebral region from L1 to L5. The resulting models were saved in STL format files. Geomagic Studio 12 (Geomagic Inc., North Carolina, USA) was used for wrapping, smoothing, and solidifying. Hypermesh (Altair Technologies, Fremont, CA, USA) was used to mesh and construct the structures of the intervertebral disc, bone, and ligaments. All simulations were conducted using Ansys Workbench 2021 (ANSYS Inc., USA).

**Fig. 3:**
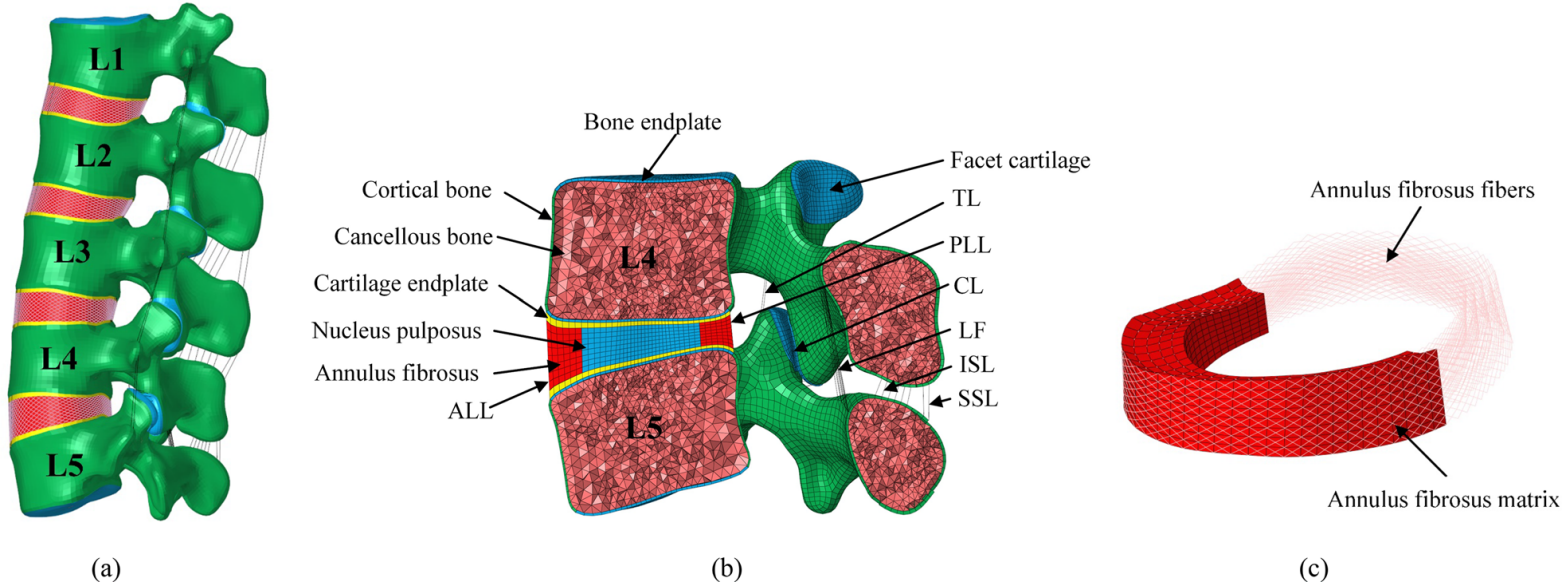
The FEM of the lumbar spine L1–L5 segment. **a** The lateral view; **b** The section view of L4–L5 segment; **c** Distribution of annulus fibrosus fibers

The intact L1–L5 lumbar finite element model consists of cortical bone, bone endplate, cancellous bone, cartilage endplate, intervertebral disc, and ligaments (Fig. 3). Cortical bone, bone endplate, cancellous bone, and cartilage endplate was defined as linear elastic materials, the intervertebral disc, and facet cartilage were modeled as nearly incompressible hyperelastic materials. The intervertebral disc has two main components: the annulus fibrosus and the nucleus pulposus. The nucleus pulposus constitutes 43% of the intervertebral disc [36]. The annulus fibrosus was similar to the reinforced concrete structure, composed of the annulus fibrosus matrix and fibers. The fibers were embedded in the annulus fibrosus matrix, and the angle between the fibers and the endplate surface was about ± 30° [37]. The thickness of cortical bone is about 1 mm, and the initial gap between the two facet cartilage surfaces was about 0.1 mm. Their interaction was defined as surface-to-surface contact with zero friction coefficient [38]. Each segment incorporated simulations of seven ligaments, namely, the anterior longitudinal ligament (ALL), posterior longitudinal ligament (PLL), ligamentum flavum (LF), capsular ligament (CL), inter-transverse ligament (ITL), interspinous ligament (ISL), and supraspinous ligament (SSL) [39]. The annulus fibers and ligaments meshed using nonlinear truss elements without compression [40]. The material properties were determined based on previously reported literature, as presented in Table 1 [41–45].

**Table 1:** Material properties of the lumbar FEM

Component name	Young’s modulus (MPa)	Poisson’s ratio	Cross-section area (mm^2^)
Cortical bone	12,000	0.3	–
Cancellous bone	100	0.3	–
Bone endplate	12,000	0.3	–
Cartilage endplate	25	0.3	–
Facet cartilage	Neo-Hookean, C10 = 2		–
Annulus ground	Mooney-Rivlin, C1 = 0.12, C2 = 0.03		–
Nucleus pulposus	Mooney-Rivlin, C1 = 0.18, C2 = 0.045		–
ALL	7.8 (< 12.0%)	0.3	63.7
	20 (> 12.0%)		
PLL	10 (< 11.0%)	0.3	14.4
	20 (> 11.0%)		
LF	15 (< 6.2%)	0.3	40
	19.5 (> 6.2%)		
ISL	10 (< 14.0%)	0.3	26
	11.6 (> 14.0%)		
SSL	8 (< 20.0%)	0.3	23
	15 (> 20.0%)		
TL	10 (< 18.0%)	0.3	1.8
	58.7 (> 18.0%)		
CL	7.5 (< 25.0%)	0.3	30
	32.9 (> 25.0%)		

### Finite element postoperative model

**Fig. 4:**
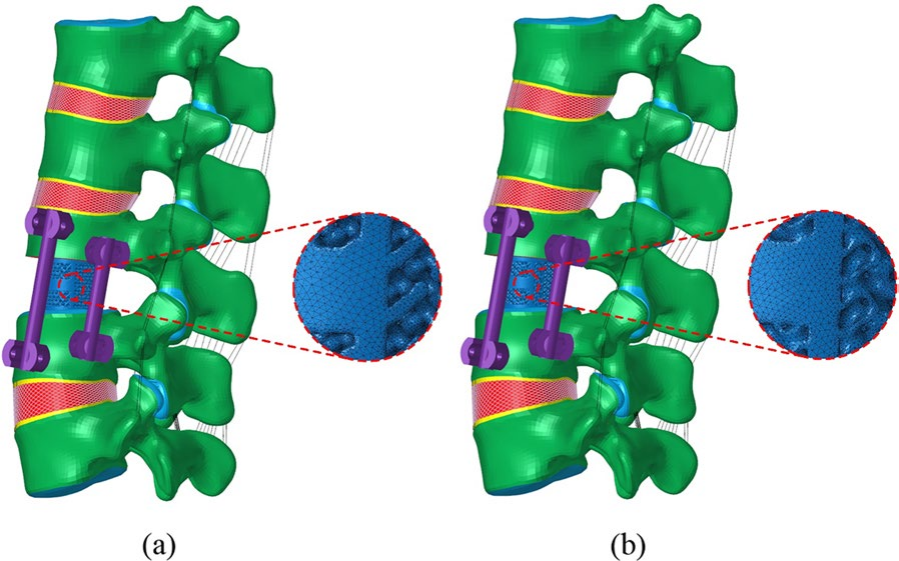
Prosthetic reconstruction models. **a** Model A; **b** Model B

The internal fixator, pedicle screws (6.0 × 40 mm), and rods (5.5 mm) were built by SolidWorks (Dassault Systems, Paris, France). In this study, two surgical finite element models were created (Fig. 4). The lower third of the L3 vertebrae and the L3/L4 disc were removed in all models and fixed laterally using two pairs of pedicle nails. In model A, the prosthetic is a traditional porous AVI; in model B, the prosthetic is a Gyroid porous AVI. The material properties in the operated model are presented in Table 2 [46]. For all FEMs, geometric matching at the prosthesis-endplate interface was achieved using the “Boolean calculation” to remove the portion of the AVI that overlapped with the vertebral body. To accurately analyze the biomechanical effects of the two porous AVI, the mesh of the porous structures was refined. Rigid connections were formed among bone and screws, screws, and rods to mimic the fastening conditions, and a ‘Bonded’ contact was made between the bone and prosthetic to mimic the healed phase [17, 47, 48].

**Table 2:** Material properties of operation model

Component name	Young’s modulus (MPa)	Poisson’s ratio
Titanium	110,000	0.3
Bone grafts	100	0.2

### Boundary and loading conditions

Ansys Workbench 2021 was employed to establish boundary and load conditions and simulate spinal movement. The L5 vertebral body was assumed to be immobile, with its substructure as a fixed boundary with no displacement or rotation in any direction. To simulate flexion, extension, lateral bending, and torsion load conditions, a 400-N uniform load and a 10-Nm moment were applied to the upper surface of the L1 vertebra [49, 50].

### Sample preparation

The samples were fabricated using a selective laser melting machine (LiM-X150A, LiM Laser, China) and Ti6Al4V powder, which meets the ISO 5832-3 standard. The printing parameters are shown in Table 3.

**Table 3:** Main printing parameters of LiM-X150A

Laser power/W	Scanning distance/mm	Layer thickness/mm	Scanning speed/(mm/s)
170	0.09	0.03	1250

**Fig. 5:**
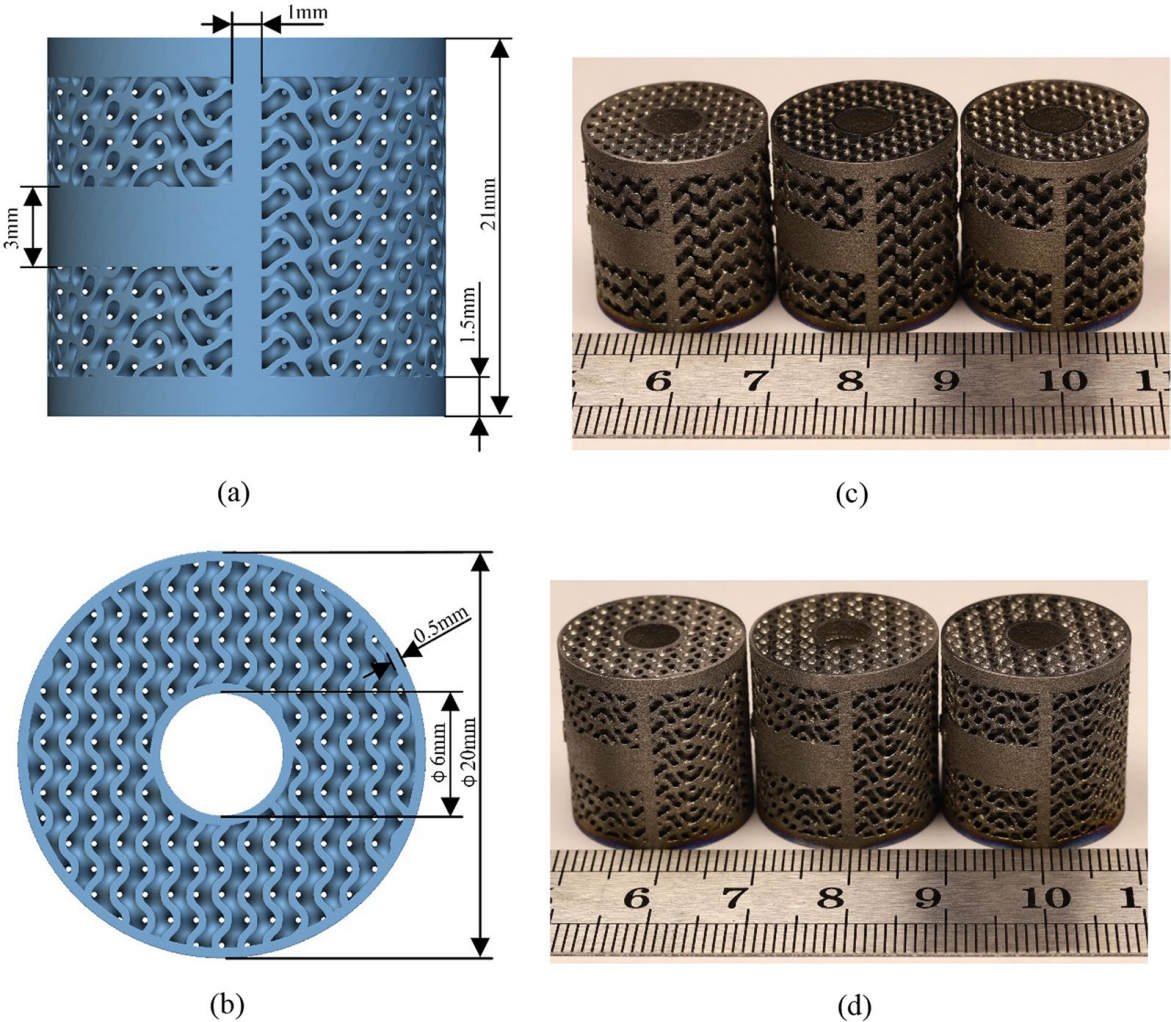
Detailed information of porous AVI specimens. **a** and **b** Geometric parameters of Gyroid porous AVI; **c** Traditional porous AVI specimens; **d** Gyroid porous AVI specimens

When the 3D printing process was completed, the samples were heat treated in an oven at 920 °C for 4 h to eliminate internal stress. Subsequently, the substrates were removed, and the samples were meticulously cleaned in an ultrasonic cleaner operating at 37 kHz for 60 min to eliminate any residual powder particles from the surface. Afterward, the samples were dried for 60 min. Three replicates of each sample type were printed (Fig. 5).

### Mechanical experiments

Static compression experiments were performed according to ASTM F2077-2014 standards to determine the maximum compression load for all specimens. All specimens (*N* = 3) were axially compressed in a universal testing machine (WDW-200Y) operating at a 1 mm/min loading rate of up to 10 mm compressive displacement. The load-to-displacement ratio was continuously recorded until implant failure occurred, defined as either plastic deformation or sample fracture.

Static subsidence experiments were performed according to the ASTM F2267-04 standard, with the testing machine loaded at a rate of 6 mm/min. Load versus displacement was recorded until reaching a 3 mm displacement. The load required for 3 mm subsidence of all samples in a test block made of Grade 15 polyurethane foam was obtained.

## Results

### Validation of the model

**Fig. 6:**
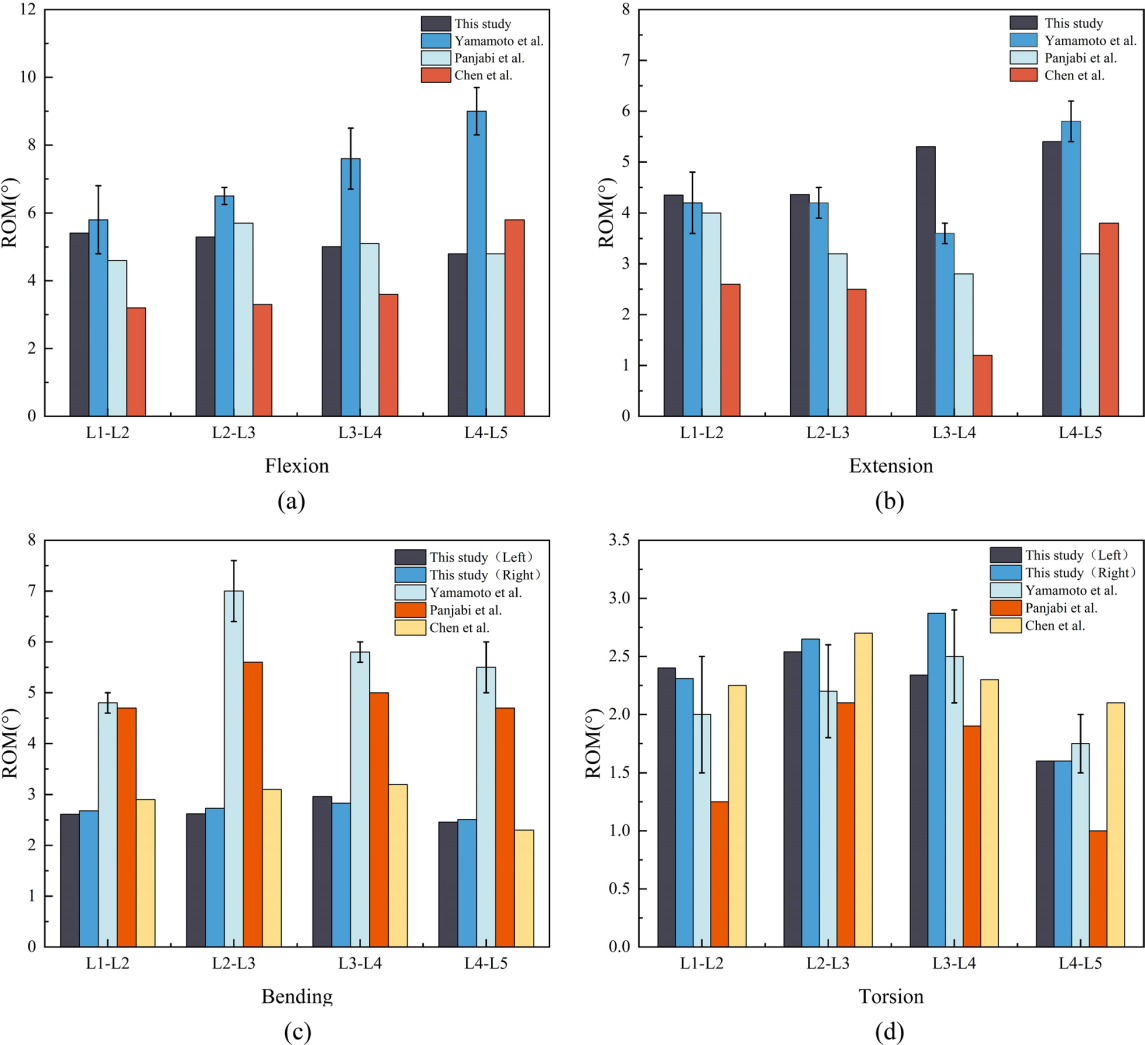
Comparison between ROM values from the lumbar spine model in this study and previously reported values. **a** Flexion; **b** Extension; **c** Bending; **d** Torsion

Figure 6 compares the range of motion (ROM) for the L1–L5 segment obtained in this study with previously published data from biomechanical and finite element analysis experiments assessing flexion, extension, bending, and torsion [51–53]. The ROM of each vertebra was close to the results of human specimens and existing finite element analysis, thus validating the current model.

### Von Mises stress of the bone-prosthetic interface vertebrae

**Fig. 7:**
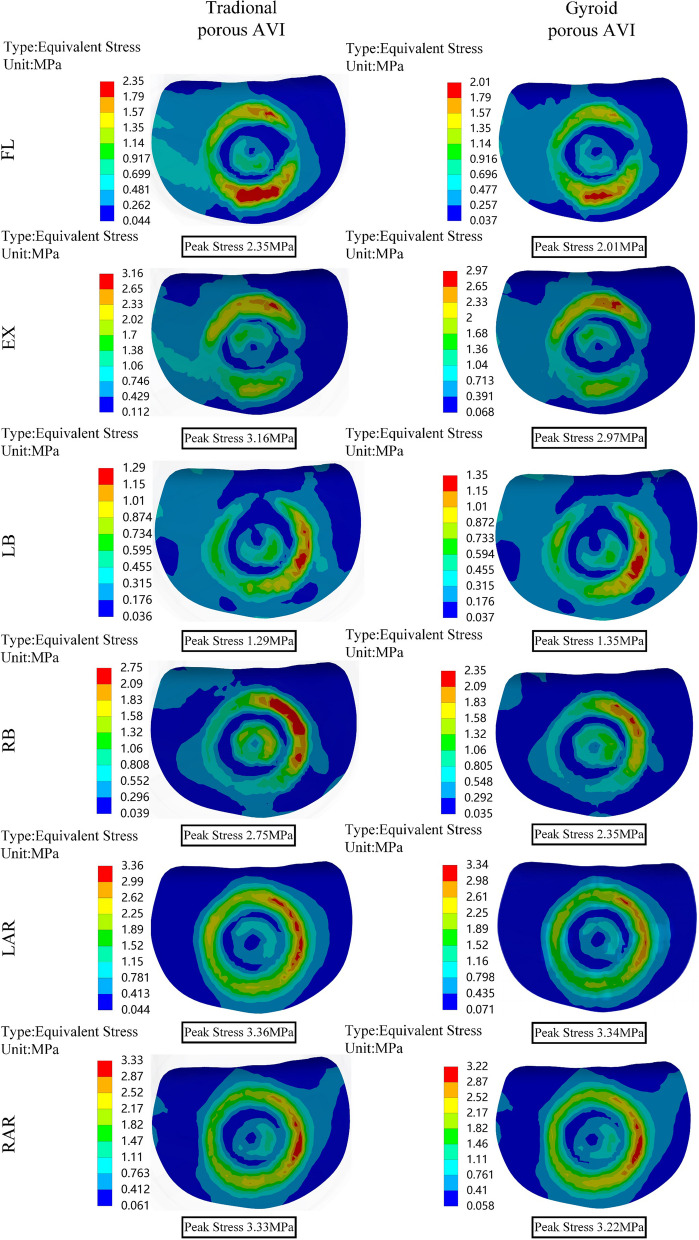
Von Mises stress (MPa) distribution of the vertebrae at the upper adjacent interface. *FL* flexion, *EX* extension, *LB* left lateral bending, *RB* right lateral bending, *LAR* left axial rotation, *RAR* right axial rotation

**Fig. 8:**
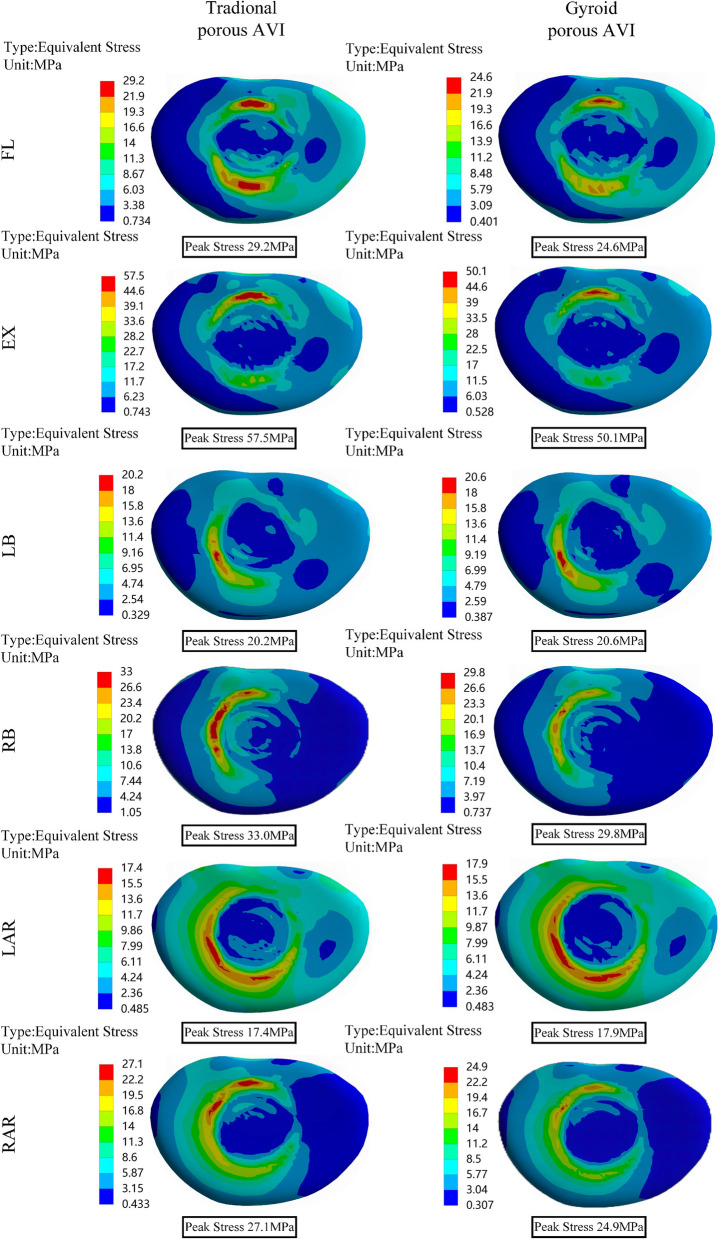
Von Mises stress (MPa) distribution of the vertebrae at the lower adjacent interface

Stress concentration areas were observed in the contact region between the prosthesis and the vertebrae, as shown in Figs. 7 and 8. The peak stress of the bone-Gyroid porous AVI interface vertebrae, was lower than that of the bone-traditional porous AVI interface vertebrae, except for the left bending and left torsion conditions specifically, during forward flexion, the peak stress of the vertebrae at the upper adjacent interface decreased by 14.5%, extension by 6.0%, right lateral bending by 14.5%, and right lateral rotation by 3.3%. Similarly, at the lower adjacent interface, the peak stress decreased by 15.5% during forward flexion, 12.9% during extension, 9.7% during right lateral bending, and 8.1% during right lateral rotation.

### Von Mises stress of the prosthetic

**Fig. 9:**
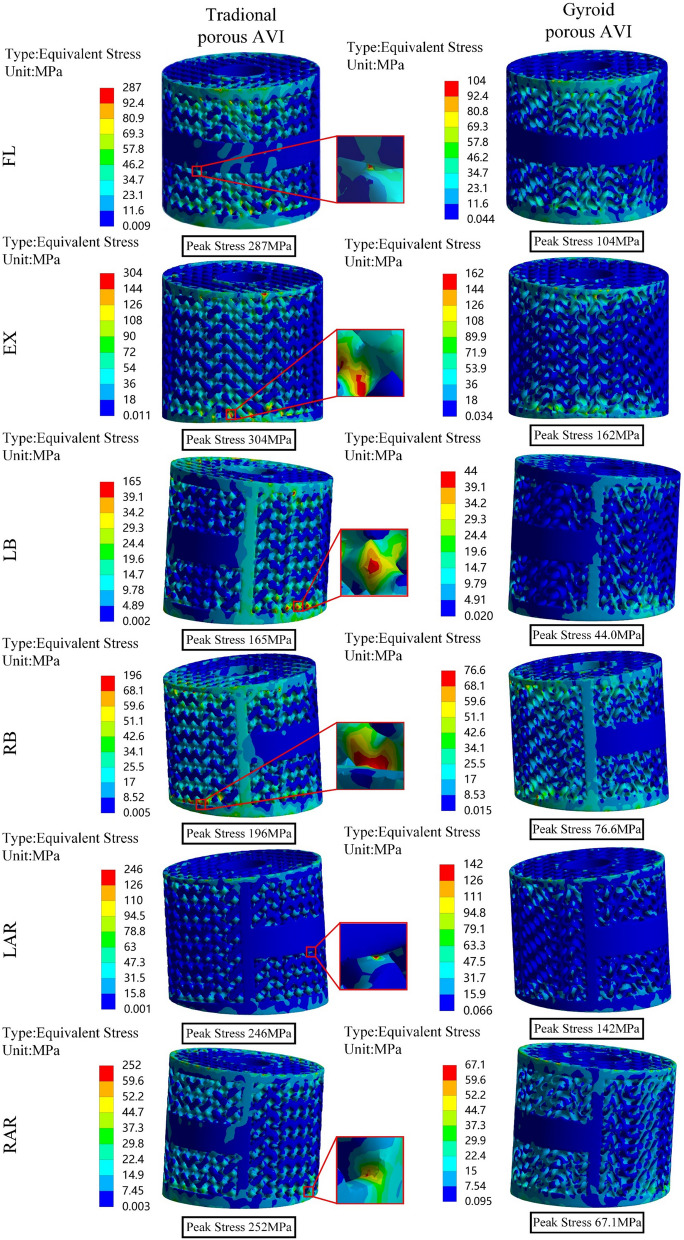
Von Mises stress (MPa) distribution of the porous AVIs

As shown in Fig. 9, stress distribution on both the traditional porous AVI and the Gyroid porous AVI is associated with loading conditions. Significant differences in peak stress were observed among the two prostheses. The Gyroid porous AVI exhibited significantly lower peak stress compared to the traditional porous AVI, with a decrease in 63.8% in forward flexion, 46.8% in extension, 73.4% in left lateral bending, 61.0% in right lateral bending, 42.1% in left lateral rotation, and 72.4% in right lateral rotation. The traditional porous AVI stress concentration occurred at the junction between the porous and frame structures. In contrast, the Gyroid porous AVI was distributed more uniformly. Notably, no significant stress concentrations were at the joints with the frame.

### Porosity measurement and analysis

**Fig. 10:**
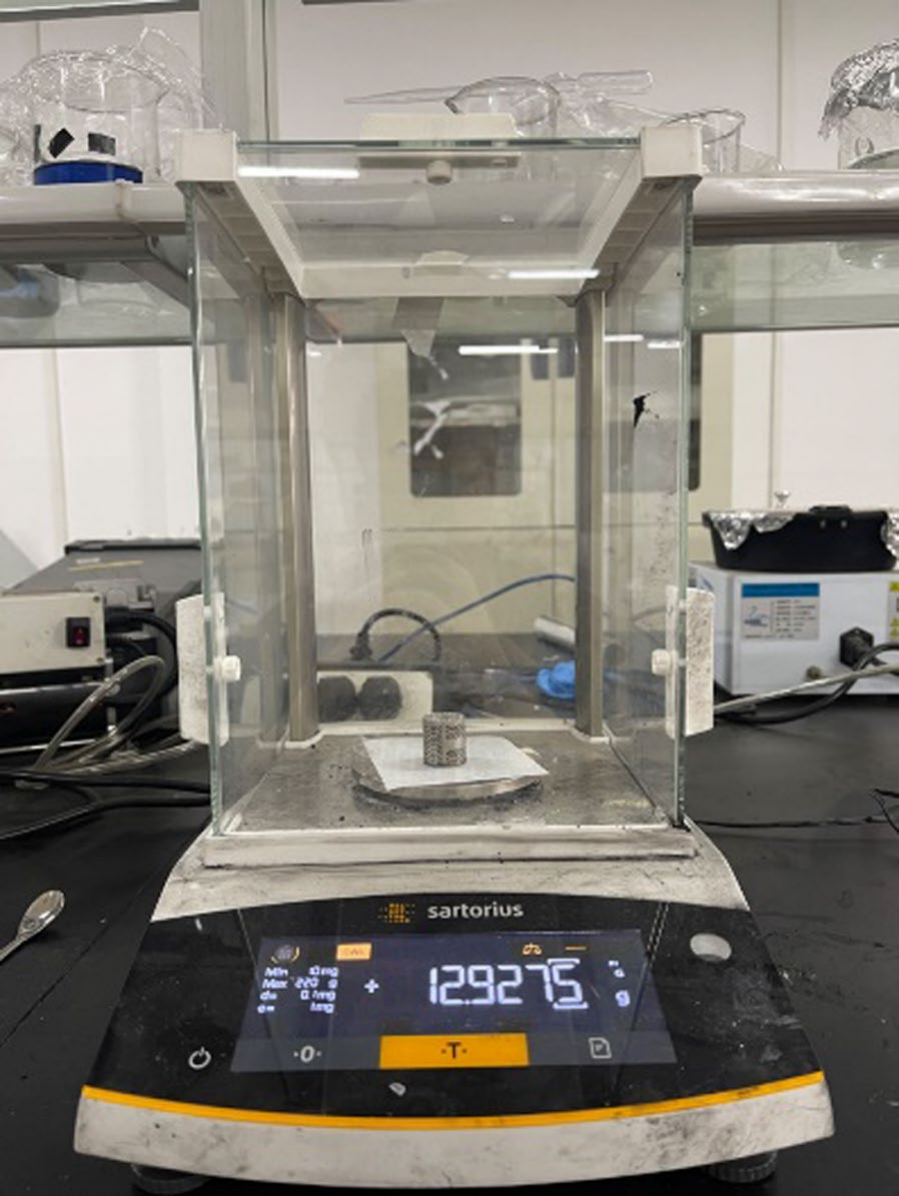
Electronic balance weighing

An electronic balance (BCE224-1CCN) measured each specimen's mass, as shown in Fig. 10. Subsequently, the specimen's porosity can be calculated using the following equation.

2 $$P = \left( {1 - \frac{{m_{{\text{p}}} - m_{{\text{k}}} }}{{m_{{\text{s}}} - m_{{\text{k}}} }}} \right) \times 100\%$$

where *P* is the porosity of porous structures inside AVI specimens, *m*_p_ is the mass of AVI specimens (g), *m*_s_ is the mass of solid Ti–6Al–4V specimens (g), and *m*_k_ is the mass of AVI solid frames (g).

**Fig. 11:**
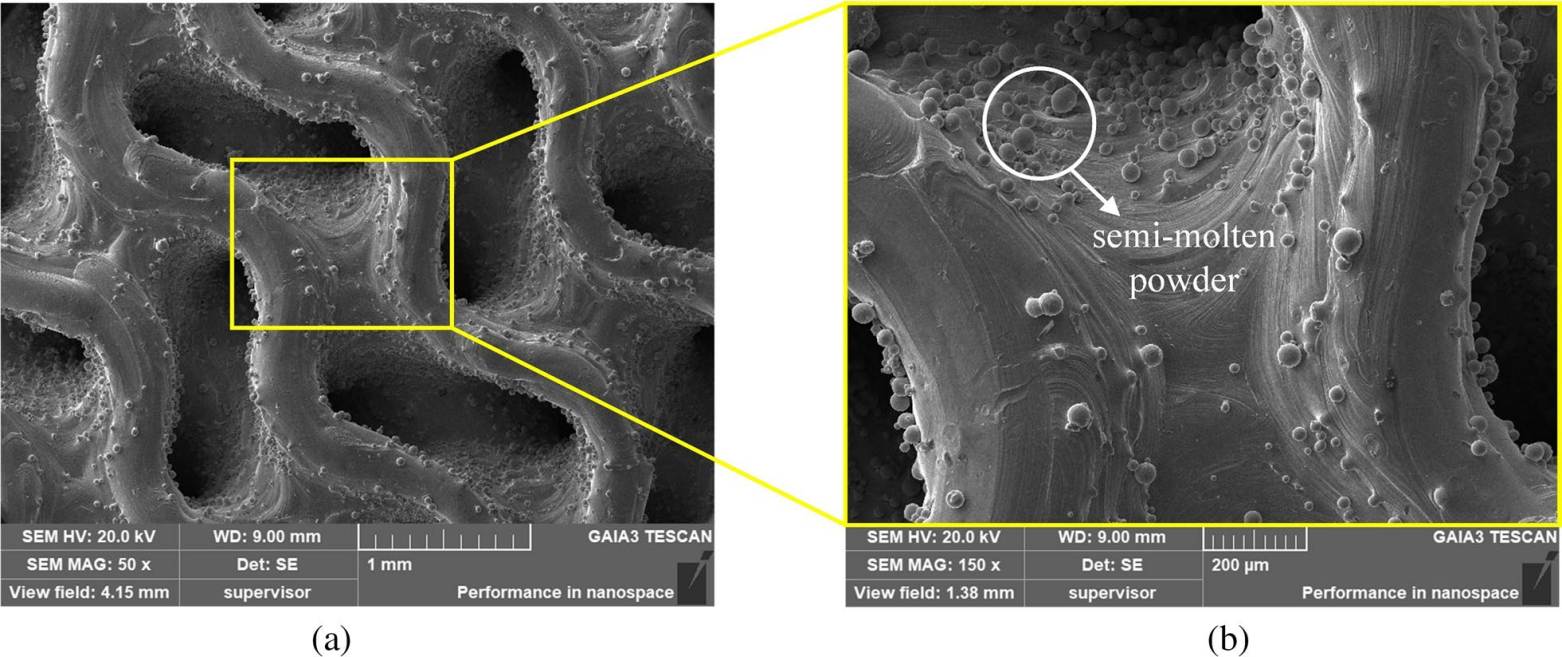
SEM images presenting. **a** The upper surface of a Gyroid porous AVI specimen. **b** Semi-molten powder on the specimen surface

The porosity of the AVI specimens was generally within 3% of the target, which was acceptable [54]. The deviation of the porosity may be due to the adhesion of the semi-molten powder on the specimen surface (Fig. 11), and the measured porosity of all the specimens was less than the CAD-designed porosity (Table 4).

**Table 4:** Porosity deviation of SLM specimens from designs

Specimens	Designed porosity/%	Specimens porosity/%	Difference/%
Traditional porous AVI	60	58.9 ± 0.53	1.83
Gyroid porous AVI	60	59.4 ± 0.41	1.00

### Static subsidence experiments

**Fig. 12:**
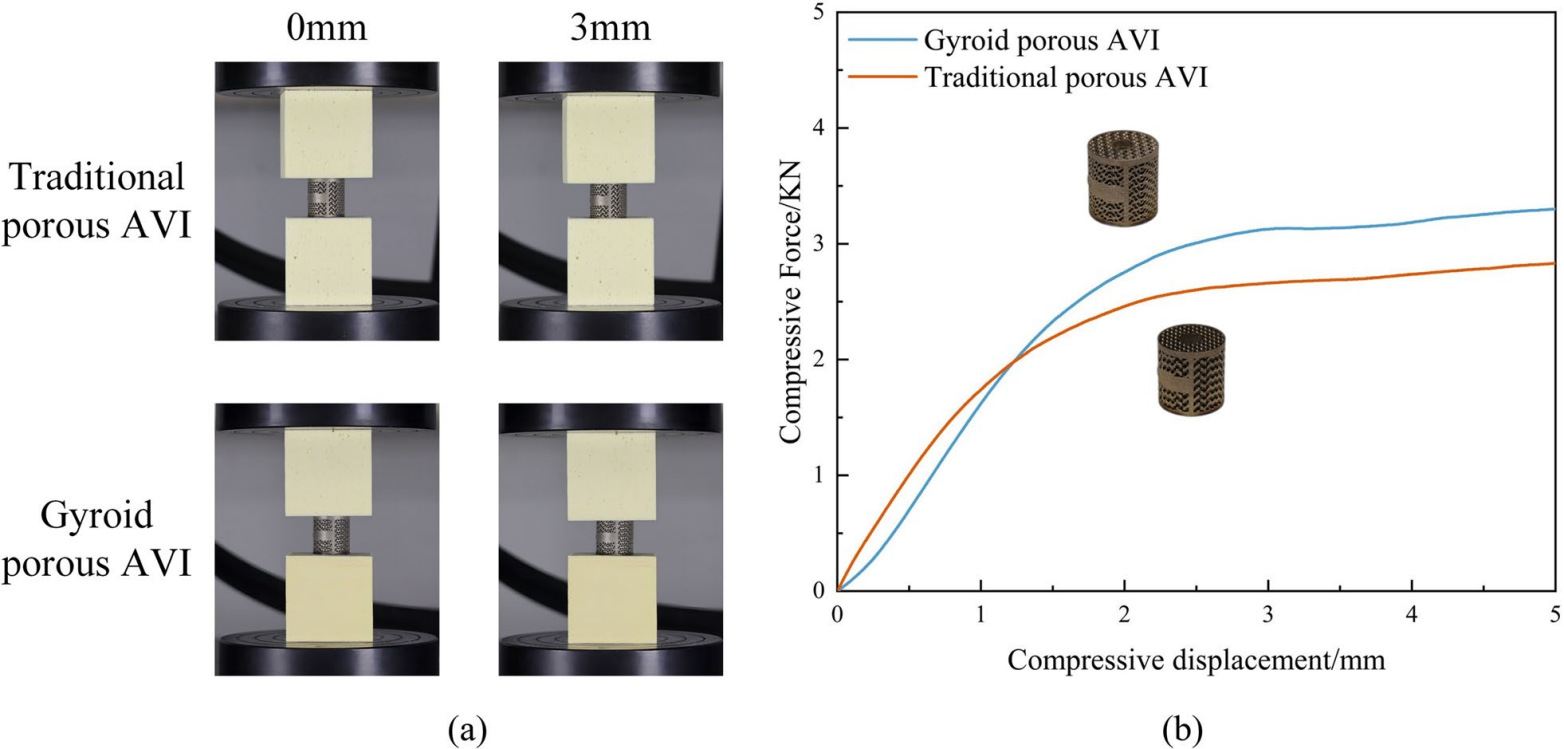
Results of static subsidence test of AVIs. **a** AVIs at different stages of subsidence displacement; **b** The force–displacement curve under the static subsidence testing

Figure 12a presents a schematic diagram of the two AVIs compressed at 0 mm and 3 mm during the static subsidence experiments. Figure 12b illustrates the relationship between load and displacement recorded in the static subsidence experiments. The results revealed that the average load recorded for the traditional porous AVI at 3 mm of subsidence was 2660 N ± 8 N, while the average load for the Gyroid porous AVI was 3158 ± 10 N. Compared to the traditional porous AVI, the average load for the Gyroid porous AVI was 15.7% higher. As a result, the tendency of Gyroid porous AVI to subside into polyurethane foam (simulated cancellous bone) was reduced by approximately 15.7%.

### Static compression experiments

**Fig. 13:**
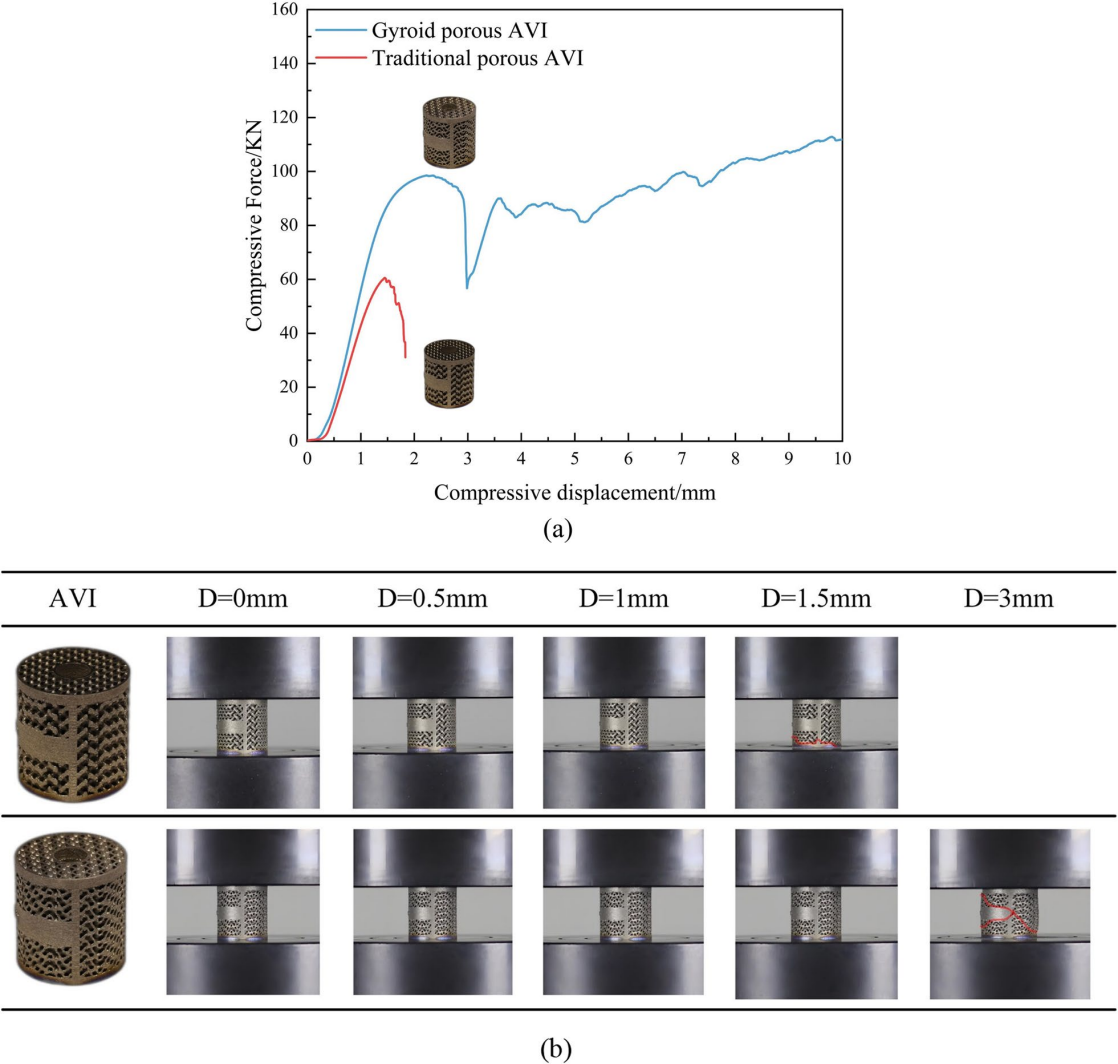
Results of compression testing of AVIs. **a** The force–displacement curve under the compressive testing; **b** Deforming process of AVIs at different stages of compression displacement

The relationship between load and displacement recorded during the static compression experiments is illustrated in Fig. 13a. In contrast, Fig. 13b displays the photographs of the recorded compression experiments utilized to analyze deformation and damage patterns. The experiment results revealed that the maximum compressive load was 60.52 ± 1.53 kN/mm for the traditional porous AVI and 98.4 ± 2.3 kN/mm for the Gyroid porous AVI. The maximum compressive load for the Gyroid porous AVI was 38.51% higher than that for the traditional porous AVI. Furthermore, the images recorded at various test stages showed no cracks in both porous AVIs until 1.5 mm of compression. However, for the traditional porous AVI, a fracture occurred between the bottom frame of the AVI and the porous structures when the compression displacement reached 1.5 mm. On the other hand, for the Gyroid porous AVI, the AVI failed at 3 mm of compression displacement, mainly manifested as a 45° shear fracture.

## Discussion

TMC reconstruction is the most frequently employed method for reconstruction after thoracolumbar laminectomy. However, the mismatch between the TMC and the endplate leads to a poor contact area, further leading to prosthesis subsidence and potential fracture occurrences [55]. In recent years, 3D printing technology has changed this situation. 3D printing technology enables prosthesis design methods to evolve from traditional standardized design to personalized design. Accurate modeling ensures perfect fitting between 3D-printed AVIs and the vertebral interface, thus helping achieve uniform stress transfer under different load conditions. At the same time, the porous structures can effectively reduce or eliminate the 'stress shielding' effect [2–4]. However, it should be noted that the porous structures will reduce the implant's safety to a certain extent [27, 56]. Furthermore, the porous structures influence the osseointegration capacity [57]. In summary, designing and implementing porous structures within the AVI is particularly important.

Strut-based porous structures, such as body-centered cubic cells (BCC), face-centered cubic cells (FCC), and diamond structures [5–7], suffer from inadequate compressive strength and propensity for stress concentration within their lattice structures [8–10]. In contrast, the TPMS structures with higher mechanical properties have significant advantages over these strut-based porous structures [8, 58]. Furthermore, the TPMS structures are better suited for promoting bioactivity and bio-integration with bone tissue because of their specific structural characteristics [59–61]. When used as implants like AVI, the TPMS structure could strengthen the integration of the implant with the surrounding bone tissue, reduce the risk of prosthesis subsidence and mechanical failure, and further enhance the implant's long-term efficiency.

The Gyroid porous AVI developed in this study fully utilizes the advantages of the TPMS structure, and its biomechanical properties were comprehensively evaluated through finite element and experimental methods. The study focused on two crucial indicators of the implant: prosthesis strength and prosthesis subsidence resistance to ensure the safety and stability of the implant within the human body. This study is expected to offer a new and practical approach to enhance prosthesis performance and reduce postoperative complications.

Excessive stress on the prosthesis can hasten its mechanical failure. In contrast, insufficient stress can impede fusion speed and compromise fusion efficiency. Therefore, the stress analysis on the prosthetic was conducted in this study. There was a pronounced stress concentration phenomenon at the connection points between the units and the frame structure in traditional porous AVI. In contrast, the peak stress of the Gyroid porous AVI was substantially reduced, ranging from 42.1 to 73.4%. This behavior can be attributed to the TPMS structure's continuously curved surface, allowing it to avoid localized stress concentrations and maintain a smooth distribution on the surrounding surfaces [62]. Furthermore, the peak stress of the Gyroid porous AVI was lower than the fatigue strength of the 3D printed solid samples (approximately 200–300 MPa) [63, 64]. In contrast, the traditional porous AVI either exceeded or approached the material's fatigue strength. These findings demonstrate that the Gyroid porous AVI exhibits excellent stress distribution capabilities and meets the safety requirements for daily activities.

Elevated interfacial and non-uniform stress distribution significantly contribute to prosthetic subsidence [65, 66]. They can also result in mechanical failure of the prosthesis [67]. Therefore, the stress in the vertebrae at the bone-prosthetic interface was examined in the study. Postoperative reconstruction using Gyroid porous AVI effectively reduces peak stress in the interface vertebrae compared to traditional porous AVI reconstruction. The peak stress at the upper adjacent interface of the vertebrae exhibited the highest reduction, approximately 14.5%. Similarly, the peak stress at the lower adjacent interface of the vertebrae showed the most substantial decrease, about 15.5%. This phenomenon can be attributed to the design of the porous structures, which effectively reduces the modulus of elasticity of the solid metal to match that of human bone tissue closely. Moreover, the increased contact area with the vertebrae promotes more uniform stress distribution, mitigating the risk of prosthesis subsidence.

Static compression experiments of two porous AVIs revealed that the Gyroid porous AVI was about 1.63 times that of traditional porous AVI in terms of the maximum compression load. Compared with the results in the literature [16, 68], the data concerning maximum compressive load proves that the structure parameters of Gyroid porous AVI also meet the safety requirements. During the compression displacement of 1.5 mm for the traditional porous AVI, conspicuous cracks emerged at the combination of the frame and the porous structures at the bottom of the AVI, consistent with the findings obtained from finite element analysis of the traditional porous AVI, which showed high stress at the interface between the porous structures and the frame. The experimental data and failure record images provided strong evidence for the superior strength and stability of the novel AVI with Gyroid porous structures. Simultaneously, the established finite element model demonstrated its efficacy in the biomechanical evaluation of the AVI.

The static subsidence experiments involving two porous AVI demonstrated that Gyroid porous AVI settled approximately 15.7% less than traditional porous AVI when tested in polyurethane foam (simulated cancellous bone). Additionally, finite element analysis revealed that Gyroid porous AVI reduced the risk of prosthetic subsidence. This benefit is likely attributable to the more extensive implant-bone interface of the Gyroid porous structures than traditional ones. The increased contact area allows for more well-distributed stress, thus lowering the risk of subsidence.

This study possesses several limitations. Firstly, the FEM modeling data obtained from individual image data may vary among individuals within the population. Furthermore, the study solely focused on a particular Gyroid design without exploring other TPMS designs. Subsequent research should assess various TPMS porous design features to enhance our understanding of optimal AVI design concepts, aiming for improved subsidence and fusion performances. Finally, the study focused on analyzing the biomechanical performance of the Gyroid porous AVI using FEM and mechanical experiments. While these methods provide valuable insights into the biomechanical properties of the implant, further clinical validation through in vivo studies and long-term patient follow-up is necessary to confirm the safety and efficacy of the implant in real-world applications.

## Conclusions

This study comprehensively evaluated the biomechanical properties of the Gyroid porous AVI using both finite element analysis and experimental methods. The finite element analysis revealed that the Gyroid porous AVI exhibited significantly lower peak stress than the traditional porous AVI, with a maximum reduction of 73.4%. Moreover, it effectively reduced the peak stress at the bone-implant interface. Additionally, mechanical testing demonstrated that the Gyroid porous AVI had higher compressive strength and lower subsidence tendency than traditional porous AVI. The thorough evaluation of the novel AVI with Gyroid porous structures demonstrates its significant advantages in biomechanical properties, suggesting it is a promising treatment option for patients in clinical applications.

## Data Availability

Please contact the corresponding author for data requests.

## Abbreviations

AVIs: Artificial vertebral implants
TPMS: Triply periodic minimal surface
FEM: Finite element method
SLM: Selective laser melting
TMC: Titanium mesh cage
ROM: Range of motion
DICOM: Digital imaging and communications in medicine
